# Inhibitors of *IFN* gene stimulators (*STING*) improve intestinal ischemia–reperfusion-induced acute lung injury by activating *AMPK* signaling

**DOI:** 10.1186/s40001-022-00703-1

**Published:** 2022-05-31

**Authors:** Mei Yang, Yu-Xia Ma, Ying Zhi, Hai-Bin Wang, Li Zhao, Peng-Sheng Wang, Jie-Ting Niu

**Affiliations:** Department of Gerontology, Cangzhou Central Hospital, Hebei Medical University, No. 16, Xinhua West Road, Cangzhou, China

**Keywords:** Acute lung injury, Intestinal ischemia–reperfusion, *STING*, *AMPK*, Inflammation

## Abstract

**Background:**

Acute lung injury (ALI) caused by intestinal ischemia–reperfusion is a life-threatening disease. Interferon gene stimulator (*STING*) is a cytoplasmic DNA sensor that participates in the initiation of the inflammatory response. This study aims to establish whether *C-176* (*STING* inhibitor) improves ALI under intestinal ischemia–reperfusion conditions.

**Methods:**

To induce ALI, 72 male C57BL/6 mice were subjected to intestinal ischemia for 60 min and reperfusion for 3 h. Through intraperitoneal injection, *C-176*, a selective *STING* inhibitor, was injected 30 min before surgical treatment; meanwhile, compound C, an antagonist of adenosine monophosphate-activated protein kinase (*AMPK*), was administered 30 min after surgery. Based on immunofluorescence and Western blot assays, post-ALI assessments included lung water content (TLW), bronchoalveolar lavage fluid (BALF) protein, H&E staining, Masson staining, pulmonary pyroptosis [Gasdermin-D (GSDMD), cleaved caspase-1], and apoptosis (TUNEL, cleaved caspase-3).

**Results:**

*C-176* administration significantly attenuated intestinal ischemia–reperfusion-mediated ALI; this effect was reflected by exacerbated TLW and BALF protein, aggravated lung injury score, elevated degree of pulmonary fibrosis, increased TUNEL- and GSDMD-positive cells, and upregulated phospho-*AMPK*, cleaved caspase-1, cleaved caspase-3 and IFNβ mRNA expression. Moreover, *C-176* increased phospho-*AMPK* under ALI conditions. Nonetheless, compound C partially reversed these beneficial effects.

**Conclusion:**

*C-176*, a selective *STING* inhibitor, improves intestinal ischemia–reperfusion-mediated ALI, and its underlying mechanism may be associated with *AMPK* signal activation.

## Introduction

Acute lung injury (ALI) is a severe respiratory disease causing high global mortality. It is characterized by dyspnea, interstitial edema, accumulation of activated inflammatory cells, mass migration of neutrophils, and diffuse alveolar damage [1, 2]. A growing body of recent evidence indicates that distant lung organ injuries, including abdominal ischemia–reperfusion, infection, and surgery, can cause ALI [3–5]. Moreover, reports indicate that the inflammatory response after intestinal ischemia–reperfusion injury promotes ALI [6]. To date, potential treatments or drugs against intestinal ischemia–reperfusion injury-induced ALI are unavailable in clinical therapy.

Interferon gene stimulator (*STING*) is an important mediator of the innate immune response; it detects double-stranded DNA (dsDNA) in the cytoplasm of immune cells, including DCs, T cells, and macrophages [7]. After catalyzing endogenous dsDNA leaking from mitochondria and exogenous dsDNA from pathogens by cyclic *GMP-AMP* synthase (*cGAS*), *STING* translocates from the endoplasmic reticulum to perinuclear microsomes via the Golgi apparatus [8]. Notably, *STING* activation controls mitochondrial DNA-mediated lung injury by evoking an inflammatory storm [9]. Several studies indicate that lipopolysaccharide (LPS)-induced ALI is associated with upregulation of *STING* expression [10, 11]. To date, limited studies have explored *STING* function in intestinal ischemia–reperfusion injury-induced ALI.

Although *STING* is essential in the process of ALI [12, 13], its downstream proteins remain underexplored. Notably, adenosine monophosphate-activated protein kinase (*AMPK*) promotes various anabolic and catabolic signals, hence maintaining suitable levels of adenosine triphosphate under energetic and/or cellular stress [14]. Interestingly, the *STING–AMPK* signal is implicated in high-fat diet-induced cardiac anomalies [15]. Moreover, compound C, which is annotated as a reversible inhibitor of *AMPK*, inhibits dsDNA-dependent type I interferon induction [16]. However, the role of *STING–AMPK* signaling in the ALI model should be further investigated.

This study hypothesizes that surgical intervention induces intestinal ischemia–reperfusion followed by ALI. Using a selective inhibitor of *STING* and an *AMPK* inhibitor, we aimed to determine the role of *STING–AMPK* signaling in the pathogenesis of ALI after intestinal ischemia–reperfusion injury.

## Materials and methods

Adult male C57/BL mice (7–9 weeks old; weight, 27.1 ± 2.3 g) were purchased from Changsheng Biotechnology Co., Ltd. (Benxi, Liaoning, China). All the mice had free access to food and water; they were kept in a 12-h alternating light and dark facility at 25 °C ± 1 °C (humidity 50–70%). The tests involving animals were performed following the guidelines of the Animal Ethics Committee of Cangzhou Central Hospital.

### Grouping and intestinal ischemia–reperfusion (IR)-induced ALI

In the first stage, animals were randomly grouped into five groups: sham (*n* = 24); intestinal ischemia–reperfusion (IR) + vehicle (*n* = 24); IR + C-176 (350 nmol, 4.7 mg/kg) (*n* = 18); IR + C-176 (550 nmol, 7.3 mg/kg) (*n* = 24) and IR + C-176 (750 nmol, 10 mg/kg) (*n* = 18). In the second stage, animals were randomly divided into two groups: IR + C176 + compound C (*n* = 12) and IR + C176 + Vehicle (*n* = 12).

**Fig. 1:**
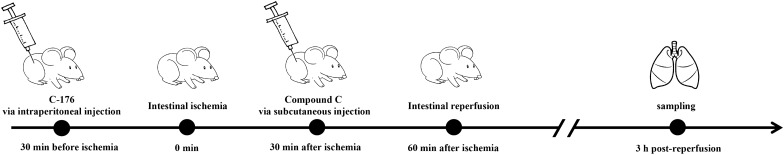
Experimental schematic diagram. Mice with intestinal ischemia–reperfusion injury (IR) and *C-176* treatment. IR: mice received an occlusion of the superior mesenteric artery below the celiac trunk with an arterial microclamp for 60 min and reperfusion for 3 h. *C-176*: *C-176* administered via intraperitoneal injection 30 min before surgical exposure. Compound C: compound C was administered via subcutaneous injection 30 min after the initiation of IR. Vehicle: 10% DMSO and 90% corn oil were administered via intraperitoneal or subcutaneous injection as a control. Sham: mice subjected to identical surgery and vehicle injection except for the occlusion of the superior mesenteric artery

Mice were anesthetized with pentobarbital (10 mg/kg) via intraperitoneal injection. An oral endotracheal tube for the mouse was established and then connected with a ventilator (model: volume-controlled; tidal volume: 8 mL/kg; frequency: 120 beats/min). Throughout the experiment, the animals were kept on a warm blanket to maintain their body temperature within 37–38 °C. Based on previous studies [17], the IR model was established as follows: (1) a lower midline laparotomy was performed; (2) the superior mesenteric artery was identified and occluded below the celiac trunk with an arterial microclamp, and intestinal ischemia was confirmed by the paleness of the jejunum and ileum; (3) the clamp was removed after 60 min; (4) 0.5 mL of sterile saline at 37 °C was injected into the peritoneal cavity; and (5) the incision was sutured and blocked using ropivacaine. In the first stage, a selective *STING* inhibitor, three dosages of C-176 (4.7 mg/kg, 7.3 mg/kg, and 10 mg/kg) (HY-112906, MedChemExpress, NJ, USA) were administered 30 min before surgical exposure via intraperitoneal injection. In the pd stage, a selective antagonist of *AMPK*, compound C (25 mg/kg) (HY-13418A, MedChemExpress), was administered via subcutaneous injection 30 min after initiation of IR. Both *C-176* and compound C were dissolved with 10% DMSO (HY-Y0320, MedChemExpress) and 90% corn oil (HY-Y1888, MedChemExpress). The mice in the sham, IR + Vehicle, and IR + C176 + Vehicle groups were only administered solvent containing 10% DMSO and 90% corn oil using intraperitoneal or subcutaneous injection. Notably, the sham animals underwent a similar procedure without clamps (Fig. 1).

### Assessment of lung injury

Three hours after reperfusion, mice (*n* = 6) were euthanized through cervical dislocation under 8% sevoflurane. The surface of the left lungs was wiped after rinsing with saline. The weight was recorded as wet weight (W). After drying at 70 °C for 24 h, the dry weight (D) was recorded. Total lung water content (TLW) was calculated using the equation TLW = (W − D)/D × 100%.

Mice (n = 6) were anesthetized under 3–4% sevoflurane and injected with 0.5 mL of normal saline via the trachea. Then, the liquid of bronchoalveolar lavage was gently aspirated, and bronchoalveolar lavage was performed twice. After centrifugation at 4000×*g* at 4 ℃ for 10 min, the supernatant from the lavage solution was obtained as bronchoalveolar lavage fluid (BALF). The content of BALF protein was measured using the BCA assay based on the manufacturers’ instructions (P0012, Beyotime, Shanghai, China).

### Measurement of diamine oxidase (DAO) and IL-1β in serum

After 3 h of reperfusion, mice (*n* = 6) were anesthetized under 3–4% sevoflurane. Blood was collected from a ventriculus sinister. Then, the mice were perfused with cold saline via the ventriculus sinister–aorta until clear fluid flowed out from the right atrial appendage. Lung tissues were quickly kept on ice. The lung tissues for RNA and protein were removed and homogenized on ice. Based on DAO synthesized primarily in gastrointestinal mucosal cells, the integrity and functional mass of the intestinal mucosa can be reflected by the level of serum DAO [18]. IL-1β, a marker of the inflammatory response in the serum, was also used to validate the distant organ injury primarily caused by circulating mediators in blood. After centrifugation at 1200×*g* for 10 min, the serum from the blood sample was used to evaluate DAO and IL-1β levels. According to the manufacturer’s protocol, we detected the level of serum DAO via a chemical assay kit (Cat# A088-1-1, Nanjing Jiancheng Bioengineering Institute, Nanjing, China) with a spectrophotometer. The results were expressed as units per liter serum. Serum IL-1β levels were evaluated by ELISA according to the manufacturer's protocol (Cat# PI301, Beyotime).

### Real-time PCR

Total RNA was extracted with a TRIeasy™ Plus Total RNA Kit (Cat# 19211ES60, Yeasen, China) according to the manufacturer's protocol. The extracted RNA (400 ng) was treated with DNase I and then reverse-transcribed into cDNA by a BeyoRT™ Q First Strand cDNA Synthesis Kit (Beyotime) according to the manufacturer’s protocol. Real-time PCR was performed using the Step One Real-Time PCR system (ABI). The expression levels of RNA were normalized to β-actin. The sequences of the primers used for real-time PCR were:

β-actin: 5′-TTTGCAGCTCCTTCGTTGC-3′ (F).

5′-TCGTCATCCATGGCGAACT-3′ (R).

IFNβ: 5′-CCAGCTCCAAGAAAGGACGA(F).

5′-CGCCCTGTAGGTGAGGTTGAT-3′ (R).

### Hematoxylin and eosin (H&E) staining and Masson staining

Three hours after reperfusion, mice (*n* = 6) were anesthetized under 3–4% sevoflurane and perfused with cold saline via the ventriculus sinister–aorta. Mice were perfused with 10% paraformaldehyde after clear saline was released from the right auricle. After fixing with 10% paraformaldehyde for 48 h, the left lungs were cut into 5-μm paraffin coronal sections for hematoxylin and eosin (H&E) and Masson staining as previously published [19, 20]. The slides were observed under a light microscope (BX51; Olympus, Tokyo, Japan). Three fields (magnification,  × 200) in one slice (3 slices in one group) were randomly selected. Lung injury was analyzed by an experienced investigator blinded to the group and recorded as normal (0), mild (1), moderate (2), or severe (3) based on histological parameters, including alveolar edema, diffuse alveolar hemorrhage, congestion, and intra-alveolar infiltration of inflammatory cells. Additionally, the percentage of Masson-stained collagen was measured using ImageJ (1.37v, Wayne Rasband, available through the National Institutes of Health).

### Immunofluorescence staining

The slices (thickness: 5 μm) mentioned above were used for immunofluorescence staining. After boiling with sodium citrate at 100 °C for 20 min, the cooled sections were incubated with 1% Triton X-100 for 20 min and blocked with QuickBlock™ Blocking Buffer (P0260, Beyotime) at 25 °C for 1 h. After washing with PBS three times, the sections were incubated overnight with rabbit anti-gasdermin-D (GSDMD) (K009328P, Solarbio, Beijing, China) at 4 °C. After rising with PBS, the slices were incubated with goat anti-rabbit secondary antibodies (P0208, Beyotime) at room temperature for 1 h, coated with anti-fluorescence quenching sealing solution with DAPI (P0131, Beyotime) for 5 min and sealed. Terminal deoxynucleotide transferase deoxyuridine triphosphate (dUTP) nick end labeling (TUNEL) (C1062, Beyotime) assays were performed according to the manufacturer’s protocol to detect pulmonary apoptosis. Six fields with a magnification of × 200 in 3 slices were randomly selected from each group. Under a fluorescence microscope (BX53, Olympus, Tokyo, Japan), the percentage of GSDMD-positive cells and TUNEL-positive cells was calculated using ImageJ (1.37v, Wayne Rasband, available through the National Institutes of Health).

### Western blot

Total protein was extracted from lung tissue and quantified through the BCA assay. Each sample containing 40 μg of mixed loading buffer was boiled at 100 °C for 15 min. The sample was separated by 10% SDS-PAGE and transferred to a PVDF membrane. The membrane was incubated with QuickBlock™ blocking buffer (P0235, Beyotime) at 25 °C for 10 min and then rinsed with Western wash buffer (P0023C, Beyotime) for 5 min 3 times. Anti-rabbit *p-AMPK* (dilution: 1:500, ab133448, Abcam, Cambridge, UK), anti-rabbit AMPK (dilution: 1:1000, ab32047, Abcam, Cambridge, UK), anti-rabbit cleaved-caspase-1 (dilution: 1:500, ab179515, Abcam, Cambridge, UK), anti-rabbit cleaved-caspase-3 (dilution: 1:500, ab32351, Abcam, Cambridge, UK) and GAPDH (dilution 1:1000, K106389P, Solarbio) were used for overnight incubation of PVDF membranes at 4 ℃. After rising with Western washing buffer 3 times, the membranes were incubated with goat anti-rabbit secondary antibody (dilution 1:1000, A0562, Beyotime) at 25 ℃ for 1 h. After washing 3 times with Western washing buffer, protein bands were detected using BeyoECL Moon (P0018, Beyotime). The ratio between the gray value of the target protein and the GAPDH bands (internal reference) was calculated using ImageJ.

### Statistical analysis

All results are expressed as the means ± standard deviation (SD). Statistical analysis was performed using Student’s *t*-test or one-way analysis of variance (ANOVA) with a Tukey post hoc test for multiple comparisons. Differences with *P* values of less than 0.05 (*P* < 0.05) were considered statistically significant.

## Results

### *C-176* mitigates lung injury after intestinal ischemia–reperfusion injury

**Fig. 2:**
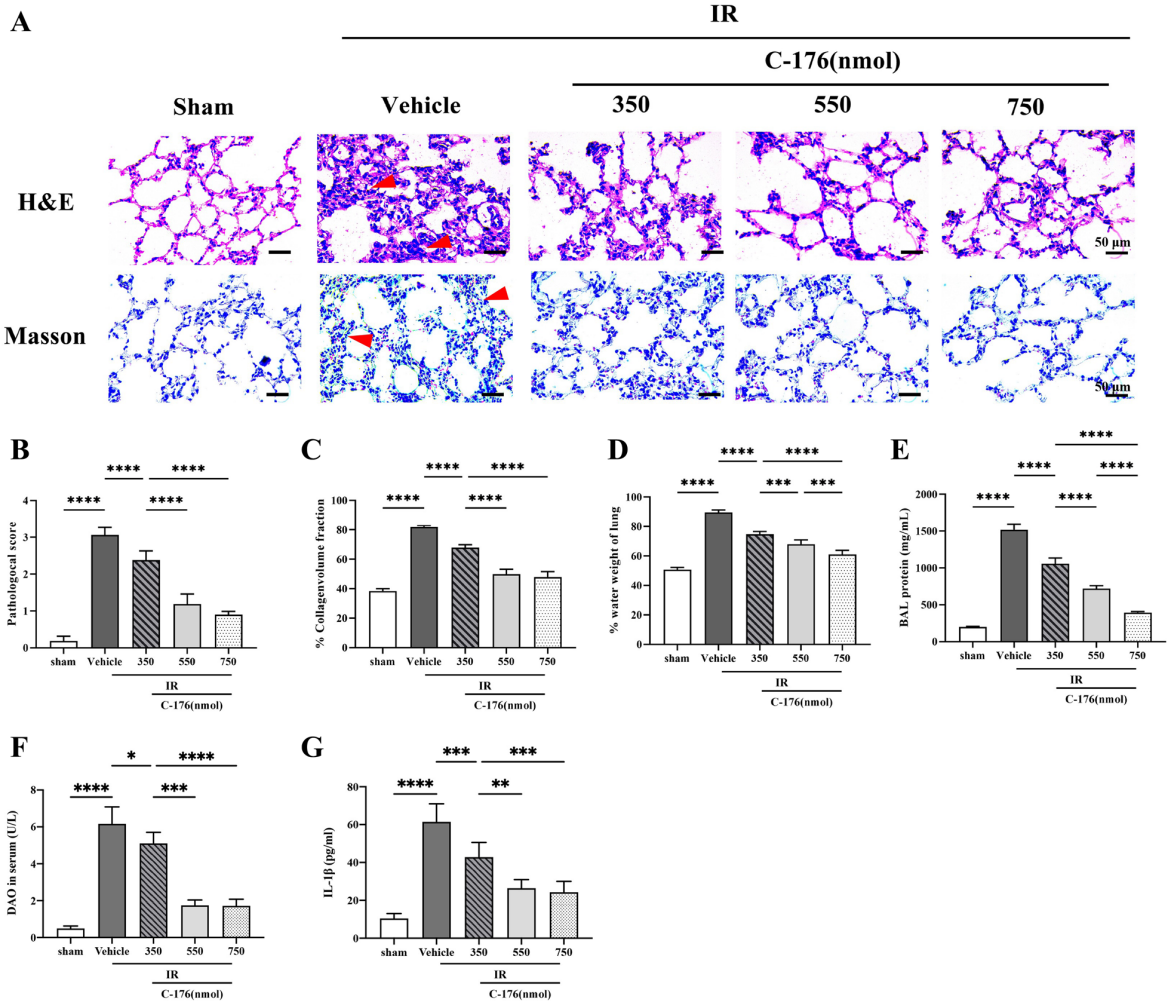
Pathological effects of *STING* on lung injury induced by IR. **A** Representative photomicrographs of H&E- and Masson trichrome-stained sections. Scale bar = 50 μm. Arrows indicate lung injury and lung collagen, respectively. **B** Lung injury score and **C** the percentage of lung collagen content caused by the indicated stimuli 3 h after IR. **D** Total lung water content (TLW); **E** content of bronchoalveolar lavage fluid (BALF) protein; **F** diamine oxidase (DAO) content in serum; and **G** IL-1β levels in the serum caused by the indicated stimuli 3 h after IR. Data are presented as the mean ± SD (*n* = 6). Sham, IR, C-176, and vehicle are described above. ****P < 0.0001, ***P < 0.001, **P < 0.01, *P < 0.05

*C-176* (a selective inhibitor of *STING*) was introduced to explore the potential role of *STING* in intestinal ischemia–reperfusion-induced ALI. Both TLW and BALF protein were used to measure pulmonary edema and exudation. Serum DAO and IL-1β were used to assess intestinal mucosa damage and circulation mediators, respectively. In addition, H&E and Masson staining were used to evaluate pulmonary structure damage and fibrosis, respectively. In contrast with the sham group, both the lung injury score (*F*_4, 25_ = 195.7, *P* < 0.0001; Fig. 2A, B) indicated by H&E staining and the percentage of collagen volume fraction (*F*_4, 25_ = 278.8, *P* < 0.0001; Fig. 2A, C) revealed by Masson staining were increased in the IR + vehicle group. Similarly, TLW (*F*_4, 25_ = 215.9, *P* < 0.0001; Fig. 2D), BALF protein (*F*_4, 25_ = 523.3, *P* < 0.0001; Fig. 2E) were significantly increased in the IR + vehicle group, and serum DAO contents (*F*_4, 25_ = 123.5, *P* < 0.0001; Fig. 2F) and IL-1β levels (*F*_4, 25_ = 54.83, *P* < 0.0001; Fig. 2G) were increased in the IR + vehicle group. Nonetheless, pathological score, the percentage of collagen volume fraction, TLW, BALF protein, serum DAO contents, and IL-1β levels were significantly decreased with increasing doses of *C-176* (Fig. 2B–G). No remarkable difference in the index mentioned above was noted between the mice exposed to intestinal ischemia–reperfusion injury with 550 nmol and 750 nmol. Thus, 550 nmol of C-176 was applied in the follow-up study.

### *C-176* mitigates pulmonary pyroptosis and apoptosis induced by intestinal ischemia–reperfusion injury

**Fig. 3:**
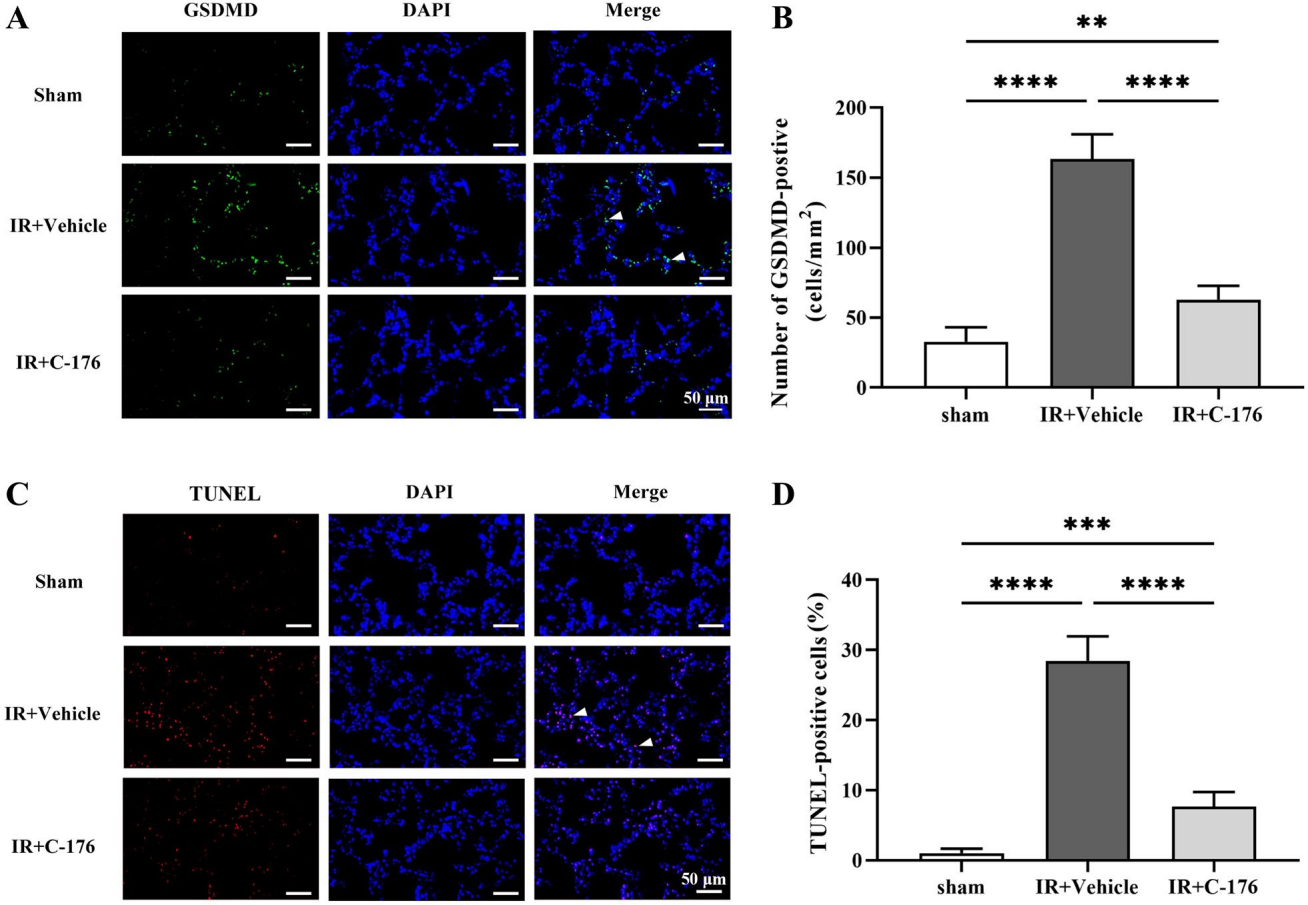
*C-176* mitigates pulmonary apoptosis and pyroptosis in mice exposed to IR. **A** Representative photomicrographs of GSDMD-positive cells; **C** TUNEL-positive cells in the lung; **B** the number of GSDMD-positive cells; and **D** the percentage of TUNEL-positive cells in the lung caused by the indicated stimuli 3 h after IR. Scale bar = 50 μm. Arrows indicate GSDMD- and TUNEL-positive cells. Data are presented as the mean ± SD (*n* = 6). Sham, IR, *C-176*, and vehicle are described above. ****P < 0.0001, ***P < 0.001, **P < 0.01

**Fig. 4:**
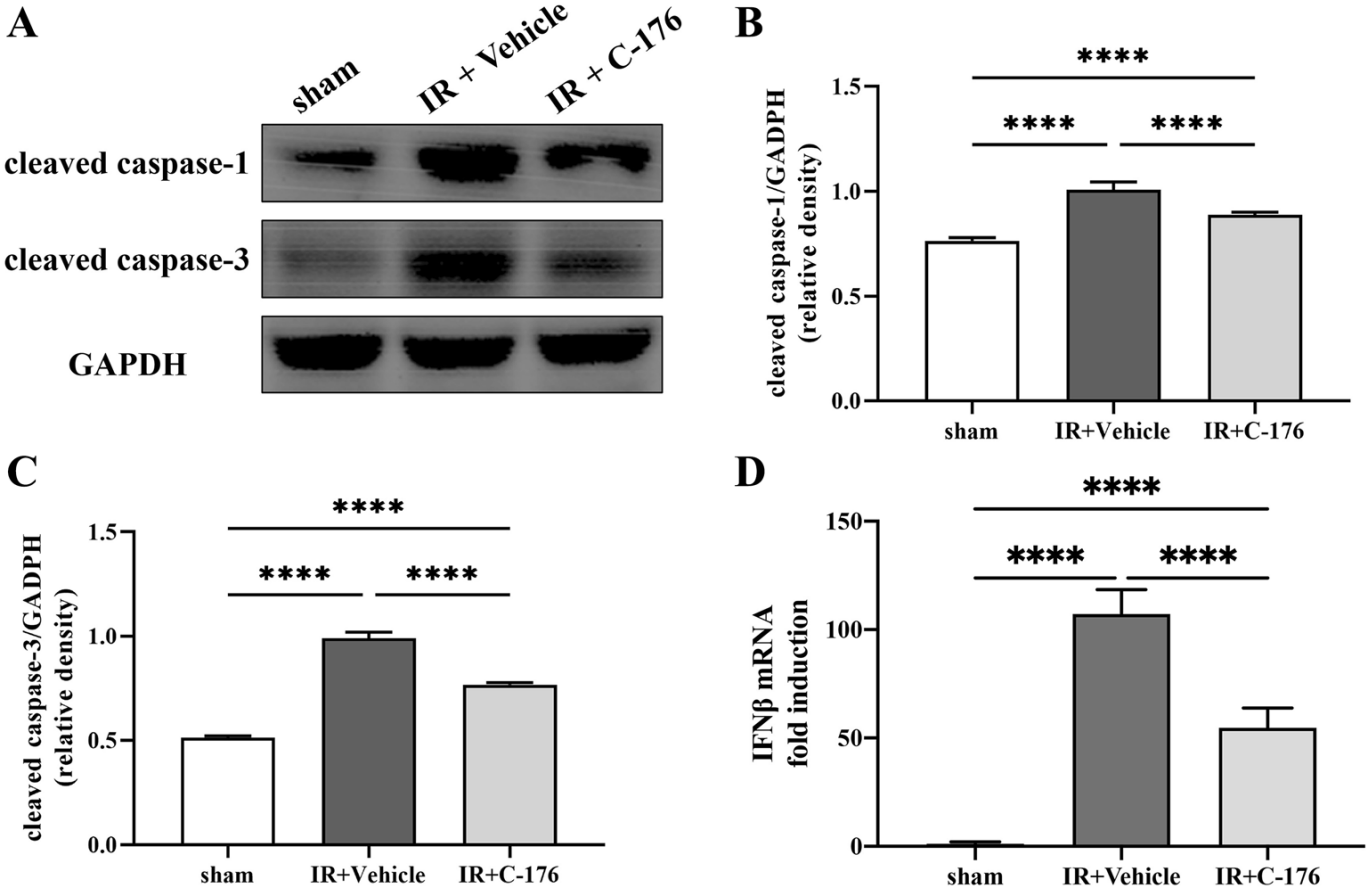
*C-176* mitigates pulmonary pyroptosis- and apoptosis-associated factors in mice exposed to IR. **A** Representative Western blot of cleaved caspase-1 (a marker for pyroptosis) and cleaved caspase-3 (a marker for apoptosis) in the lung caused by the indicated stimuli; **B** the ratio between the optical density value of cleaved caspase-1 and GAPDH in the lung, as evaluated by Western blot; **C** the ratio between the optical density value of cleaved caspase-3 and GAPDH in the lung, as evaluated by Western blot. **D** The expression of IFNβ mRNA in the lung caused by the indicated stimuli. Sham, IR, *C-176*, and vehicle are described above. ****P < 0.0001

Immunofluorescence assays for GDSMD (pyroptosis) and TUNEL (apoptosis) were used to evaluate the effects of *STING* on pulmonary pyroptosis and apoptosis induced by intestinal ischemia–reperfusion injury. As a pyroptosis execution protein, GSDMD is usually used to explore the classical pyroptosis activated by cleaved caspase-1 [21]. During the late stages of apoptosis, DNA degradation in the nuclei can be detected by the TUNEL assay [22]. Immunofluorescence showed an increased number of GSDMD-positive (*F*_2, 15_ = 162.4, *P* < 0.0001; Fig. 3A, B) and TUNEL-positive (*F*_2, 15_ = 217.1, *P* < 0.0001; Fig. 3C, D) cells in the IR + Vehicle group compared to the sham group. Nonetheless, unlike the IR + Vehicle group, both GSDMD-positive cells (*F*_2, 15_ = 217.1, *P* < 0.0001; Fig. 3A, B) and TUNEL (*F*_2, 15_ = 162.4, *P* < 0.0001; Fig. 3C, D) were significantly decreased in the IR + C-176 group. Western blot results showed that the expression levels of the apoptosis-associated factor cleaved caspase-3 (*F*_2, 15_ = 1082, *P* < 0.0001; Fig. 4A, B) and the pyroptosis-associated factor cleaved caspase-1 (*F*_2, 15_ = 148.3, *P* < 0.0001; Fig. 4A, C) were significantly upregulated in the IR + vehicle group compared to the sham group; on the other hand, *C-176* partially reversed this upregulation in the IR + C-176 group (*F*_2, 15_ = 1082, *P* < 0.0001 for cleaved caspase-3; *F*_2, 15_ = 148.3, *P* < 0.0001 for cleaved caspase-1; Fig. 4A–C). Additionally, our data also showed that IFNβ mRNA expression was heavily elevated in the IR + Vehicle group compared to that in the sham group, while *C-176* partially reversed this elevation in the IR + C-176 group (*F*_2, 15_ = 239.5, *P* < 0.0001; Fig. 4D). The above results indicate that the *STING* inhibitor *C-176* mitigates pyroptosis, apoptosis and IFNβ expression in the lung after intestinal ischemia–reperfusion injury.

### *AMPK* signal is involved in the protective effects of *C-176* against lung injury induced by intestinal ischemia–reperfusion injury

**Fig. 5:**
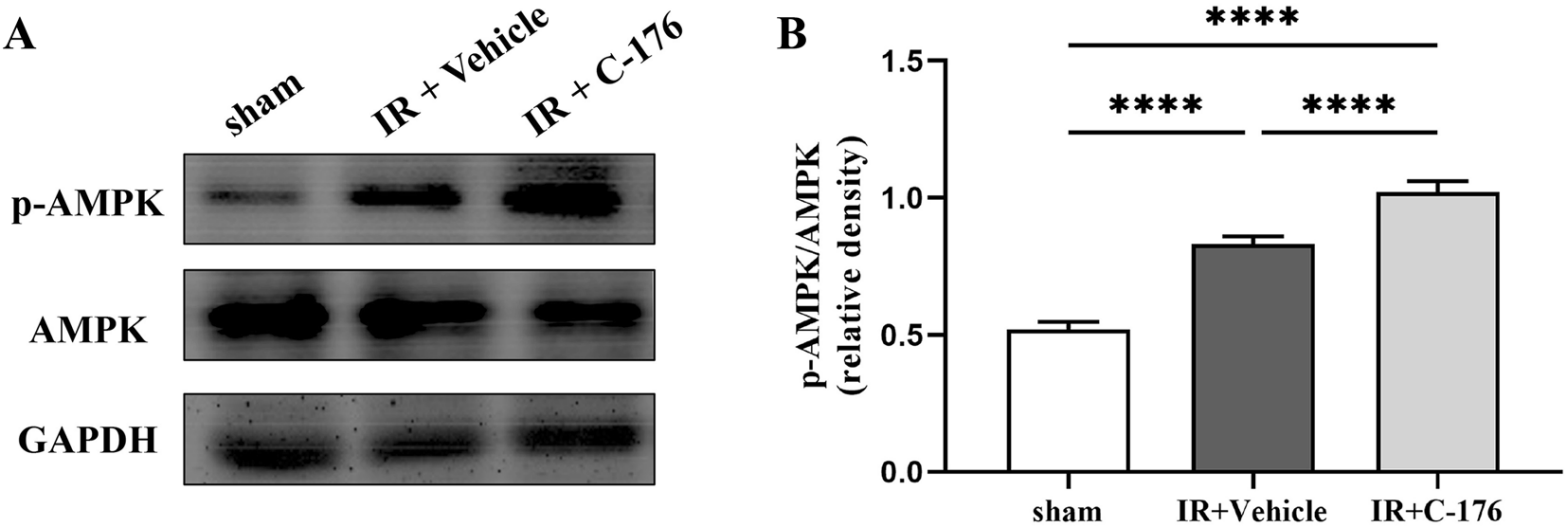
*C-176* upregulated phospho-*AMPK* expression in mice exposed to IR. **A** Representative Western blot of phospho-*AMPK* and total *AMPK* in the lung caused by the indicated stimuli; **B** the ratio between the optical density value of phospho-*AMPK* and total *AMPK* in the lung, as evaluated by Western blot. Sham, IR, *C-176*, and vehicle are described above. ****P < 0.0001

*AMPK* signaling is a downstream factor of *STING* in several processes of inflammatory injury [23, 24]. The ratio of phospho-*AMPK* to total *AMPK* was slightly increased in mice exposed to IR compared to those under sham treatment (IR + Vehicle vs. sham, *F*_2, 15_ = 348.6, *P* < 0.0001; Fig. 5A, B). Interestingly, the ratio of phospho-*AMPK* to total *AMPK* was further upregulated in mice exposed to IR plus *C-176* treatment compared to those exposed to IR plus vehicle (IR + C-176 vs. IR + Vehicle, *F*_2, 15_ = 348.6, *P* < 0.0001; Fig. 5A, B). These findings show that *AMPK* signaling may regulate the protective effects of *C-176* against lung injury induced by IR.

### Compound C, an *AMPK* inhibitor, reversed the protective effect of *C-176* on ALI

**Fig. 6:**
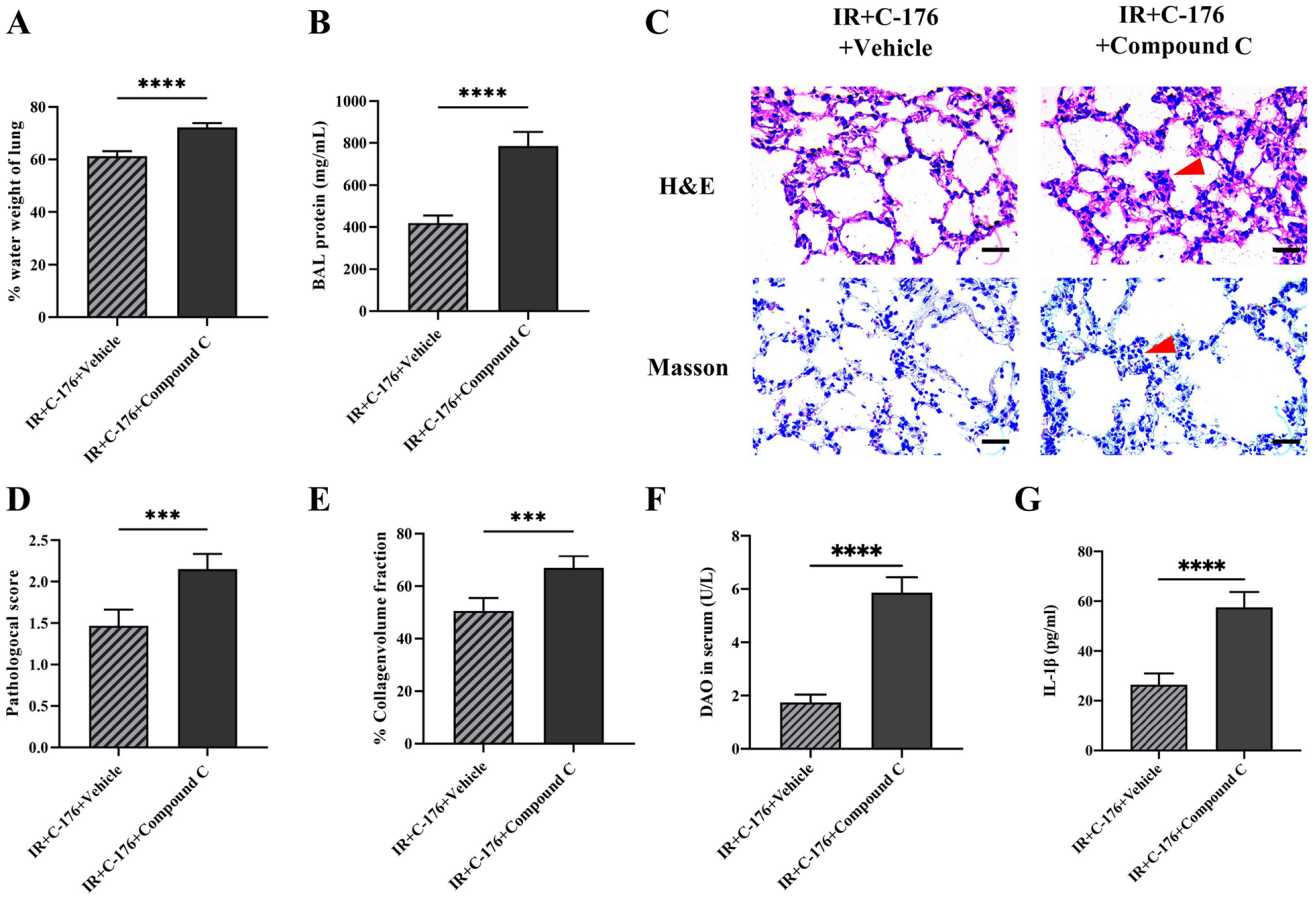
Compound C reverses the protective effects against ALI in mice exposed to IR. **A** Total lung water content (TLW). **B** Content of BALF protein caused by the indicated stimuli 3 h after IR. **C** Representative photomicrographs of H&E and Masson trichrome-stained sections. Scale bar = 50 μm; arrows indicate lung injury and lung collagen, respectively. **D** Lung injury score; **E** the percentage of lung collagen content; **F** diamine oxidase (DAO) content in serum; and **G** IL-1β levels in the serum caused by the indicated stimuli 3 h after IR. Data are presented as the mean ± SD (*n* = 6). IR, C-176, compound C and vehicle are described above. ****P < 0.0001, ***P < 0.001

**Fig. 7:**
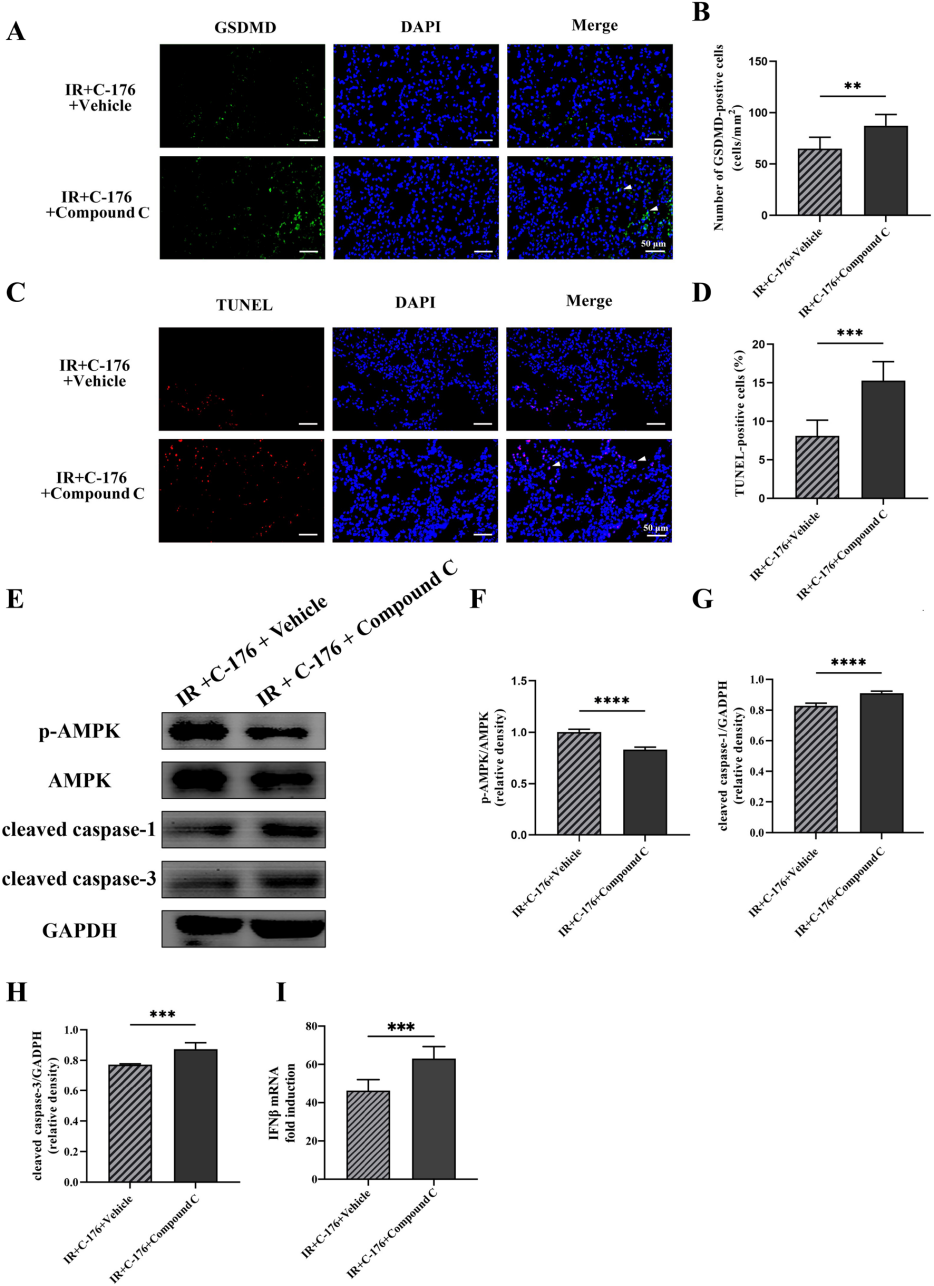
Compound C reverses the anti-pyroptotic and anti-apoptotic effects of *C-176* against ALI in mice exposed to IR. **A** Representative photomicrographs of GSDMD-positive cells and **C** TUNEL-positive cells in the lung. **B** The number of GSDMD-positive cells and **D** the percentage of TUNEL-positive cells in the lung caused by the indicated stimuli 3 h after IR. Scale bar = 50 μm. **E** Representative Western blot of phospho-*AMPK*, total *AMPK*, cleaved caspase-1 and cleaved caspase-3 in the lung caused by the indicated stimuli. **F** The ratio between the optical density value of phospho-*AMPK* and total *AMPK* in the lung, as evaluated by Western blot. **G** The ratio between the optical density value of cleaved caspase-1 and GAPDH in the lung, as evaluated by Western blot; **H** the ratio between the optical density value of cleaved caspase-3 and GAPDH in the lung, as evaluated by Western blot. **I** The expression of IFNβ mRNA in the lung caused by the indicated stimuli. Data are presented as the mean ± SD (*n* = 6). Sham, IR, *C-176*, and vehicle are described above. ****P < 0.0001, ***P < 0.001, **P < 0.01

Compound C (a selective inhibitor of *AMPK*) was introduced to further detect the role of *STING–AMPK* signaling in lung injury induced by intestinal ischemia–reperfusion injury. Unlike the IR + *C-176* + vehicle group, mice in the IR + *C-176* + compound C group showed a significant increase in TLW (*t* = 10.85, *P* < 0.0001; Fig. 6A); an elevation of BALF protein content (*t* = 11.86, *P* < 0.0001; Fig. 6B); an aggravation of lung injury score (*t* = 6.167, *P* < 0.001; Fig. 6C, D); an upregulation in the percentage of collagen volume (*t* = 6.005, *P* < 0.001; Fig. 6C, E); an increase in serum DAO content (*t* = 15.56, *P* < 0.0001; Fig. 6F); and an elevation of IL-1β in serum (*t* = 9.926, *P* < 0.0001; Fig. 6G). Additionally, GSDMD- and TUNEL-positive cells in the lung were both increased in the IR + *C-176* + compound C group relative to the IR + *C-176* + vehicle group (*t* = 3.543, *P* < 0.01 for GSDMD; *t* = 5.478, *P* < 0.001 for TUNEL; Fig. 7A–D). Based on the Western blot results, compound C significantly decreased the ratio of phospho-*AMPK*/total *AMPK* in the IR + *C-176* + compound C group relative to the IR + *C-176* + vehicle group (*t* = 11.52, *P* < 0.0001; Fig. 7E, F). Moreover, the mRNA expression levels of the pyroptosis-associated factor cleaved caspase-1 (*t* = 8.959, *P* < 0.0001; Fig. 7E, G), apoptosis-associated factor cleaved caspase-3 (*t* = 6.078, *P* < 0.001; Fig. 7E, H), and IFNβ (*t* = 4.814, *P* < 0.001; Fig. 7I) were significantly upregulated in the IR + Vehicle group compared to the sham group.

## Discussion

The present report details *STING–AMPK* signaling in the pathological process of ALI after intestinal ischemia–reperfusion injury. The main findings included the following: (1) the *STING C-176* inhibitor significantly reduced lung injury and pulmonary fibrosis after intestinal ischemia–reperfusion injury; (2) the *STING C-176* inhibitor significantly ameliorated pulmonary apoptosis and pyroptosis; and (3) the protective effects of *C-176* against ALI after intestinal ischemia–reperfusion injury may be associated with *STING–AMPK* signaling.

Endogenous toxins released from intestinal bacteria can shift to the circulatory system after intestinal ischemia–reperfusion injury, resulting in systemic inflammation, including lung injury [25]. In addition to endogenous toxins, inflammatory factors, including *IL-1* and *IL-6,* produced during ischemia enter the circulatory system [26]. In the current study, we showed that serum DAO and IL-1β were significantly increased after reperfusion, which indicates that circulation mediators were released from intestinal tissue. Relevant studies revealed that distant lung injury occurs during the process of reperfusion after intestinal ischemia; however, the mechanism of lung injury after intestinal ischemia–reperfusion injury remains unclear [27, 28]. The data showed that lung injury indicated by TLW, BALF protein content, pathological score, and collagen volume fraction were significantly aggravated after intestinal ischemia–reperfusion injury. This implies that this rodent model of intestinal ischemia–reperfusion injury potentially triggers distant lung injury, which can destroy the alveolar membrane.

The formation and activation of the inflammasome are facilitated by the *cGAS–STING* pathway, subsequently causing pyroptosis and apoptosis [29, 30]. Recent studies have reported that the inflammasome response after bacterial and viral infections is ameliorated by inhibiting the *cGAS–STING–NLRP3* axis in human myeloid cells [10]. Benmerzoug et al. reported that *STING*-mediated self-dsDNA sensing regulates the process of silica-induced lung inflammation [31]. The *cGAS–STING–NLRP3* axis in the cytoplasm is a potential therapeutic target against ALI [32]. *C-176*, which is different from another inhibitor of *STING,* such as *C-178* binding to Cys91, can block palmitoylation induced by *STING* activation [8]. *STING* assembly into polymer complexes in the Golgi apparatus is inhibited, subsequently blocking downstream signal transduction [33]. *C-176*, an effective *STING* covalent inhibitor, has been suggested to inhibit the activation of the *STING* downstream pathway and exert a robust anti‐inflammatory effect in previous studies [16, 34]. Interestingly, *STING* inhibition through a selective inhibitor (*C-176*) significantly attenuated pulmonary inflammation and fibrosis in mice induced by graphitized multiwalled carbon nanotubes [35]. As previously described, ALI is suggested to be induced by intestinal ischemia–reperfusion injury within 3 h after reperfusion [36, 37]. Mitochondrial injury-induced activation of inflammatory factors and calcium overload can reportedly contribute to cleavages of the caspase family, including caspase-1 and caspase-3, at the early stage of reperfusion, which are indicators of pyroptosis and apoptosis [38, 39]. We showed that *C-176* effectively reduced pulmonary pyroptosis and apoptosis caused by intestinal ischemia–reperfusion injury and mitigated ALI at 3 h after reperfusion. Considering the role of *C-176*, our results confirm the activation of *STING* signaling in ALI after intestinal ischemia–reperfusion injury.

A recent publication reported that the *STING–TBK1* complex inhibits phosphorylation of *AMPK*, thereby enhancing the inflammatory response in *vivo* and in vitro [40]. There is overwhelming evidence that STING can downregulate *AMPK* phosphorylation, but pharmacological inhibition of STING alleviates inflammatory injury via phosphorylated *AMPK*-related anti-inflammatory signaling [16]. Additionally, *AMPK/SIRT1* activation protects ALI induced by LPS by inhibiting pulmonary apoptosis, as indicated by a reduction in cleaved caspase-3 [41]. Endotoxin-induced ALI correlates with pyroptosis via the *AMPK/NLRC4* pathways [42]. Moreover, it was suggested that activation of *AMPK* phosphorylation ameliorates the alleviation of caspase-1-associated pyroptosis in in vivo and in vitro models of diabetic cardiomyopathy [43]. Notably, caspase-1-associated pyroptosis and caspase-3-associated apoptosis can reportedly contribute to the process of ALI after intestinal ischemia–reperfusion injury [6, 17]. We found a slight increase in *AMPK* phosphorylation, as indicated by the ratio of phosphorylated *AMPK* to total *AMPK,* in pulmonary tissue after intestinal ischemia–reperfusion injury; however, *C-176* further increased phospho-*AMPK*. Intriguingly, compound C, an inhibitor of *AMPK* phosphorylation, significantly decreased phospho-*AMPK*, partially eliminating the protective effects of *C-176*. These data indicate that *STING–AMPK* signaling is implicated in the process of ALI after intestinal ischemia–reperfusion injury.

In conclusion, we evaluated the role of *STING–AMPK* signaling in the pathophysiological process after ALI induced by intestinal ischemia–reperfusion injury. The findings support the fact that *STING–AMPK* signaling is a potentially novel therapeutic approach for the treatment of intestinal ischemia–reperfusion injury-induced ALI.

## Data Availability

All datasets generated for this study are included in the article. The datasets used and/or analyzed during the present study are available from the corresponding author on reasonable request.

## References

[1] Butt Y, Kurdowska A, Allen TC (2016). Acute lung injury: a clinical and molecular review. Arch Pathol Lab Med.

[2] Hughes KT, Beasley MB (2017). Pulmonary manifestations of acute lung injury: more than just diffuse alveolar damage. Arch Pathol Lab Med.

[3] Li Y, Cao Y, Xiao J, Shang J, Tan Q, Ping F, Huang W, Wu F, Zhang H, Zhang X (2020). Inhibitor of apoptosis-stimulating protein of p53 inhibits ferroptosis and alleviates intestinal ischemia/reperfusion-induced acute lung injury. Cell Death Differ.

[4] Islam D, Huang Y, Fanelli V, Delsedime L, Wu S, Khang J, Han B, Grassi A, Li M, Xu Y (2019). Identification and modulation of microenvironment is crucial for effective mesenchymal stromal cell therapy in acute lung injury. Am J Respir Crit Care Med.

[5] Deshpande R, Zou C (2020). *Pseudomonas*
*aeruginosa* induced cell death in acute lung injury and acute respiratory distress syndrome. Int J Mol Sci.

[6] Tan Y, Zuo W, Huang L, Zhou B, Liang H, Zheng S, Jia W, Chen S, Liu J, Yang X (2020). Nervilifordin F alleviates intestinal ischemia/reperfusion-induced acute lung injury via inhibiting inflammasome and mTOR pathway. Int Immunopharmacol.

[7] Wu J, Dobbs N, Yang K, Yan N (2020). Interferon-independent activities of mammalian STING mediate antiviral response and tumor immune evasion. Immunity.

[8] Haag SM, Gulen MF, Reymond L, Gibelin A, Abrami L, Decout A, Heymann M, van der Goot FG, Turcatti G, Behrendt R (2018). Targeting STING with covalent small-molecule inhibitors. Nature.

[9] Liu Q, Wu J, Zhang X, Li X, Wu X, Zhao Y, Ren J (2021). Circulating mitochondrial DNA-triggered autophagy dysfunction via STING underlies sepsis-related acute lung injury. Cell Death Dis.

[10] Ning L, Wei W, Wenyang J, Rui X, Qing G (2020). Cytosolic DNA-STING-NLRP3 axis is involved in murine acute lung injury induced by lipopolysaccharide. Clin Transl Med.

[11] Comish PB, Liu MM, Huebinger R, Carlson D, Kang R, Tang D (2021). The cGAS-STING pathway connects mitochondrial damage to inflammation in burn-induced acute lung injury in rat. Burns.

[12] Balka KR, Louis C, Saunders TL, Smith AM, Calleja DJ, D'Silva DB, Moghaddas F, Tailler M, Lawlor KE, Zhan Y (2020). TBK1 and IKKε act redundantly to mediate STING-induced NF-κB responses in myeloid cells. Cell Rep.

[13] Balka KR, De Nardo D (2021). Molecular and spatial mechanisms governing STING signalling. FEBS J.

[14] Carling D (2017). AMPK signalling in health and disease. Curr Opin Cell Biol.

[15] Gong Y, Li G, Tao J, Wu NN, Kandadi MR, Bi Y, Wang S, Pei Z, Ren J (2020). Double knockout of Akt2 and AMPK accentuates high fat diet-induced cardiac anomalies through a cGAS-STING-mediated mechanism. Biochim Biophys Acta Mol Basis Dis.

[16] Peng Y, Zhuang J, Ying G, Zeng H, Zhou H, Cao Y, Chen H, Xu C, Fu X, Xu H (2020). Stimulator of IFN genes mediates neuroinflammatory injury by suppressing AMPK signal in experimental subarachnoid hemorrhage. J Neuroinflammation.

[17] Kim JH, Kim J, Chun J, Lee C, Im JP, Kim JS (2018). Role of iRhom2 in intestinal ischemia-reperfusion-mediated acute lung injury. Sci Rep.

[18] Zhang XY, Liu ZM, Wen SH, Li YS, Li Y, Yao X, Huang WQ, Liu KX (2012). Dexmedetomidine administration before, but not after, ischemia attenuates intestinal injury induced by intestinal ischemia-reperfusion in rats. Anesthesiology.

[19] Tang J, Xu L, Zeng Y, Gong F (2021). Effect of gut microbiota on LPS-induced acute lung injury by regulating the TLR4/NF-kB signaling pathway. Int Immunopharmacol.

[20] Song C, He L, Zhang J, Ma H, Yuan X, Hu G, Tao L, Zhang J, Meng J (2016). Fluorofenidone attenuates pulmonary inflammation and fibrosis via inhibiting the activation of NALP3 inflammasome and IL-1β/IL-1R1/MyD88/NF-κB pathway. J Cell Mol Med.

[21] Karmakar M, Minns M, Greenberg EN, Diaz-Aponte J, Pestonjamasp K, Johnson JL, Rathkey JK, Abbott DW, Wang K, Shao F (2020). N-GSDMD trafficking to neutrophil organelles facilitates IL-1β release independently of plasma membrane pores and pyroptosis. Nat Commun.

[22] Kyrylkova K, Kyryachenko S, Leid M, Kioussi C (2012). Detection of apoptosis by TUNEL assay. Methods Mol Biol.

[23] Peng Y, Zhuang J, Ying G, Zeng H, Zhou H, Cao Y, Chen H, Xu C, Fu X, Xu H (2020). Stimulator of IFN genes mediates neuroinflammatory injury by suppressing AMPK signal in experimental subarachnoid hemorrhage. J Neuroinflammation.

[24] Prantner D, Perkins DJ, Vogel SN (2016). AMP-activated kinase (AMPK) promotes innate immunity and antiviral defense through modulation of stimulator of interferon genes (STING) signaling. J Biol Chem.

[25] Qian J, Li G, Jin X, Ma C, Cai W, Jiang N, Zheng J (2020). Emodin protects against intestinal and lung injury induced by acute intestinal injury by modulating SP-A and TLR4/NF-κB pathway. Biosci Rep.

[26] Yuan B, Xiong LL, Wen MD, Zhang P, Ma HY, Wang TH, Zhang YH (2017). Interleukin-6 RNA knockdown ameliorates acute lung injury induced by intestinal ischemia reperfusion in rats by upregulating interleukin-10 expression. Mol Med Rep.

[27] Wu D, Wang J, Li H, Xue M, Ji A, Li Y (2015). Role of hydrogen sulfide in ischemia-reperfusion injury. Oxid Med Cell Longev.

[28] Jin C, Chen J, Gu J, Zhang W (2020). Gut-lymph-lung pathway mediates sepsis-induced acute lung injury. Chin Med J.

[29] Gaidt MM, Ebert TS, Chauhan D, Ramshorn K, Pinci F, Zuber S, O'Duill F, Schmid-Burgk JL, Hoss F, Buhmann R (2017). The DNA inflammasome in human myeloid cells is initiated by a STING-cell death program upstream of NLRP3. Cell.

[30] McArthur K, Whitehead LW, Heddleston JM, Li L, Padman BS, Oorschot V, Geoghegan ND, Chappaz S, Davidson S, San Chin H (2018). BAK/BAX macropores facilitate mitochondrial herniation and mtDNA efflux during apoptosis. Science.

[31] Benmerzoug S, Rose S, Bounab B, Gosset D, Duneau L, Chenuet P, Mollet L, Le Bert M, Lambers C, Geleff S (2018). STING-dependent sensing of self-DNA drives silica-induced lung inflammation. Nat Commun.

[32] Wang W, Hu D, Wu C, Feng Y, Li A, Liu W, Wang Y, Chen K, Tian M, Xiao F (2020). STING promotes NLRP3 localization in ER and facilitates NLRP3 deubiquitination to activate the inflammasome upon HSV-1 infection. PLoS Pathog.

[33] Xiaohong L, Zhenting Z, Yunjie Y, Wei C, Xiangjin X, Kun X, Xin L, Lu L, Jun L, Pin C (2022). Activation of the STING-IRF3 pathway involved in psoriasis with diabetes mellitus. J Cell Mol Med.

[34] Pham PT, Fukuda D, Nishimoto S, Kim-Kaneyama JR, Lei XF, Takahashi Y, Sato T, Tanaka K, Suto K, Kawabata Y (2021). STING, a cytosolic DNA sensor, plays a critical role in atherogenesis: a link between innate immunity and chronic inflammation caused by lifestyle-related diseases. Eur Heart J.

[35] Han B, Wang X, Wu P, Jiang H, Yang Q, Li S, Li J, Zhang Z (2021). Pulmonary inflammatory and fibrogenic response induced by graphitized multi-walled carbon nanotube involved in cGAS-STING signaling pathway. J Hazard Mater.

[36] Dong H, Qiang Z, Chai D, Peng J, Xia Y, Hu R, Jiang H (2020). Nrf2 inhibits ferroptosis and protects against acute lung injury due to intestinal ischemia reperfusion via regulating SLC7A11 and HO-1. Aging.

[37] Chen Y, Bian W, Xu B (2020). Pretreatment with dexmedetomidine alleviates lung injury in a rat model of intestinal ischemia reperfusion. Mol Med Rep.

[38] Namura S, Zhu J, Fink K, Endres M, Srinivasan A, Tomaselli KJ, Yuan J, Moskowitz MA (1998). Activation and cleavage of caspase-3 in apoptosis induced by experimental cerebral ischemia. J Neurosci.

[39] Zheng GY, Zhang C, Li ZG (2004). Early activation of caspase-1 after retinal ischemia and reperfusion injury in mice. Chin Med J.

[40] Konno H, Konno K, Barber GN (2013). Cyclic dinucleotides trigger ULK1 (ATG1) phosphorylation of STING to prevent sustained innate immune signaling. Cell.

[41] Li X, Jamal M, Guo P, Jin Z, Zheng F, Song X, Zhan J, Wu H (2019). Irisin alleviates pulmonary epithelial barrier dysfunction in sepsis-induced acute lung injury via activation of AMPK/SIRT1 pathways. Biomed Pharmacother.

[42] He Y, Xu K, Wang Y, Chao X, Xu B, Wu J, Shen J, Ren W, Hu Y (2019). AMPK as a potential pharmacological target for alleviating LPS-induced acute lung injury partly via NLRC4 inflammasome pathway inhibition. Exp Gerontol.

[43] Yang F, Qin Y, Wang Y, Meng S, Xian H, Che H, Lv J, Li Y, Yu Y, Bai Y (2019). Metformin inhibits the nlrp3 inflammasome via AMPK/mTOR-dependent effects in diabetic cardiomyopathy. Int J Biol Sci.

